# Shared Genetic Risk Factors Across Carbamazepine‐Induced Hypersensitivity Reactions

**DOI:** 10.1002/cpt.1493

**Published:** 2019-07-03

**Authors:** Paola Nicoletti, Sarah Barrett, Laurence McEvoy, Ann K. Daly, Guruprasad Aithal, M. Isabel Lucena, Raul J. Andrade, Mia Wadelius, Pär Hallberg, Camilla Stephens, Einar S. Bjornsson, Peter Friedmann, Kati Kainu, Tarja Laitinen, Anthony Marson, Mariam Molokhia, Elizabeth Phillips, Werner Pichler, Antonino Romano, Neil Shear, Graeme Sills, Luciana K. Tanno, Ashley Swale, Aris Floratos, Yufeng Shen, Matthew R. Nelson, Paul B. Watkins, Mark J. Daly, Andrew P. Morris, Ana Alfirevic, Munir Pirmohamed

**Affiliations:** ^1^ Icahn School of Medicine at Mount Sinai New York New York USA; ^2^ Sema4, a Mount Sinai Venture Stamford Connecticut USA; ^3^ Department of Molecular and Clinical Pharmacology University of Liverpool Liverpool UK; ^4^ Institute of Cellular Medicine Newcastle University Newcastle upon Tyne UK; ^5^ National Institute for Health Research (NIHR) Nottingham Biomedical Research Unit Center at the Nottingham University Hospital NHS Trust and University of Nottingham Nottingham UK; ^6^ UGC Digestivo Clinical Pharmacology Service Instituto de Investigación Biomédica de Málaga (IBIMA) Hospital Universitario Virgen de la Victoria Universidad de Málaga Málaga Spain; ^7^ Centro de Investigación Biomédica en Red de Enfermedades Hepáticas y Digestivas (CIBERehd) Madrid Spain; ^8^ Department of Medical Sciences Science for Life Laboratory Uppsala University Uppsala Sweden; ^9^ Department of Internal Medicine Landspitali University Hospital Reykjavik Iceland; ^10^ Dermatology Unit School of Medicine University of Southampton Southampton UK; ^11^ Clinical Research Unit for Pulmonary Diseases Helsinki University Central Hospital Helsinki Finland; ^12^ School of Population Sciences and Health Services Research King's College London UK; ^13^ Departiment of Medicine Vanderbilt University Medical Center Nashville Tennessee USA; ^14^ ADR‐AC GmbH Bern Switzerland; ^15^ Allergy Unit Complesso Integrato Columbus Rome Italy; ^16^ Sunnybrook Health Sciences Centre University of Toronto Toronto Ontario Canada; ^17^ Hospital Sírio Libanês São Paulo Brazil; ^18^ Department of Systems Biology Columbia University New York New York USA; ^19^ Target Sciences GSK King of Prussia Pennsylvania USA; ^20^ Eshelman School of Pharmacy University of North Carolina Institute for Drug Safety Sciences Chapel Hill North Carolina USA; ^21^ Analytic and Translational Genetics Unit Department of Medicine Massachusetts General Hospital Harvard Medical School Boston Massachusetts USA; ^22^ Department of Biostatistics University of Liverpool Liverpool UK

## Abstract

Carbamazepine (CBZ) causes life‐threating T‐cell‐mediated hypersensitivity reactions, including serious cutaneous adverse reactions (SCARs) and drug‐induced liver injury (CBZ‐DILI). In order to evaluate shared or phenotype‐specific genetic predisposing factors for CBZ hypersensitivity reactions, we performed a meta‐analysis of two genomewide association studies (GWAS) on a total of 43 well‐phenotyped Northern and Southern European CBZ‐SCAR cases and 10,701 population controls and a GWAS on 12 CBZ‐DILI cases and 8,438 ethnically matched population controls. HLA‐A*31:01 was identified as the strongest genetic predisposing factor for both CBZ‐SCAR (odds ratio (OR) = 8.0; 95% CI 4.10–15.80; *P *=* *1.2 × 10^−9^) and CBZ‐DILI (OR = 7.3; 95% CI 2.47–23.67; *P* = 0.0004) in European populations. The association with HLA‐A*31:01 in patients with SCAR was mainly driven by hypersensitivity syndrome (OR = 12.9; *P* = 2.1 × 10^−9^) rather than by Stevens‐Johnson syndrome/toxic epidermal necrolysis cases, which showed an association with HLA‐B*57:01. We also identified a novel risk locus mapping to *ALK* only for CBZ‐SCAR cases, which needs replication in additional cohorts and functional evaluation.

**Study Highlights**

**WHAT IS THE CURRENT KNOWLEDGE ON THE TOPIC?**

☑ Carbamazepine (CBZ) is associated with serious, and sometimes fatal, cutaneous and liver adverse reactions. Genomewide profiling has shown that these predisposing factors largely reside in the HLA region (HLA‐B*15:02 and HLA‐A*31:01) consistent with the immune pathogenesis.

**WHAT QUESTION DID THIS STUDY ADDRESS?**

☑ What are the genetic predisposing factors in Northern and Southern European populations for CBZ‐induced hypersensitivity reactions affecting both the skin and liver?

**WHAT DOES THIS STUDY ADD TO OUR KNOWLEDGE?**

☑ Genetic profiling confirmed that HLA‐A*31:01 predisposes to serious cutaneous adverse reaction (SCAR) in both Northern and Southern European populations. HLA‐A*31:01 also seems to predispose to CBZ‐induced liver injury. In addition, an uncommon variant in the *ALK* gene was associated with an increased risk of CBZ‐SCAR.

**HOW MIGHT THIS CHANGE CLINICAL PHARMACOLOGY OR TRANSLATIONAL SCIENCE?**

☑ Our study adds to the overwhelming evidence of the role of HLA‐A*31:01 in predisposing to a variety of CBZ hypersensitivity phenotypes and highlights the need to implement its pre‐prescription and preemptive use in clinical settings.

Carbamazepine (CBZ) is prescribed for epilepsy, trigeminal neuralgia, and bipolar disorder.^1^ In 3–10% of patients, CBZ causes a variety of hypersensitivity reactions,^2^ ranging from mild maculopapular exanthemas to hypersensitivity syndrome, drug reaction with eosinophilia and systemic symptoms (DRESS), acute generalized exanthematous pustulosis (AGEP), Stevens‐Johnson syndrome (SJS), and toxic epidermal necrolysis (TEN); the latter four phenotypes are referred to as serious cutaneous adverse reactions (SCARs). CBZ can also lead to liver injury, which can occur either as part of DRESS or in isolation (the latter is referred to as CBZ‐drug‐induced liver injury (DILI) in this paper).^3^, ^4^ These unpredictable clinical phenotypes are T‐cell‐mediated, in which CBZ and/or its metabolites bind specific HLA molecules triggering a T‐cell response.^5^

CBZ‐induced SJS/TEN is strongly associated with HLA‐B*15:02 in Han Chinese, Thais, and Malays,^6^ whereas HLA‐A*24:02 has also recently been identified as a risk factor in Han Chinese.^7^ HLA‐A*31:01 is associated with a variety of CBZ‐SCAR phenotypes and maculopapular exanthema in Japanese,^8^ Korean,^9^ and European‐descent populations.^10^ Several other HLA loci have been suggested as susceptibility loci, including HLA‐A*02:06,^8^ HLA‐B*15:11,^11^ and HLA‐B*51:01,^12^, ^13^, ^14^ but the findings have not always been replicated.^15^ Currently, we have limited knowledge of susceptibility loci for CBZ‐DILI.

The purpose of our study was to perform a meta‐analysis between our previously published CBZ hypersensitivity cohort^10^ and a newly recruited cohort in order to increase study power and further investigate (i) novel risk loci within and outside the HLA region, and (ii) the role of HLA‐A*31:01 stratifying by European subpopulations and clinical phenotypes, including SCAR and DILI.

## Results

### Case collection and population structure

**Figure 1:**
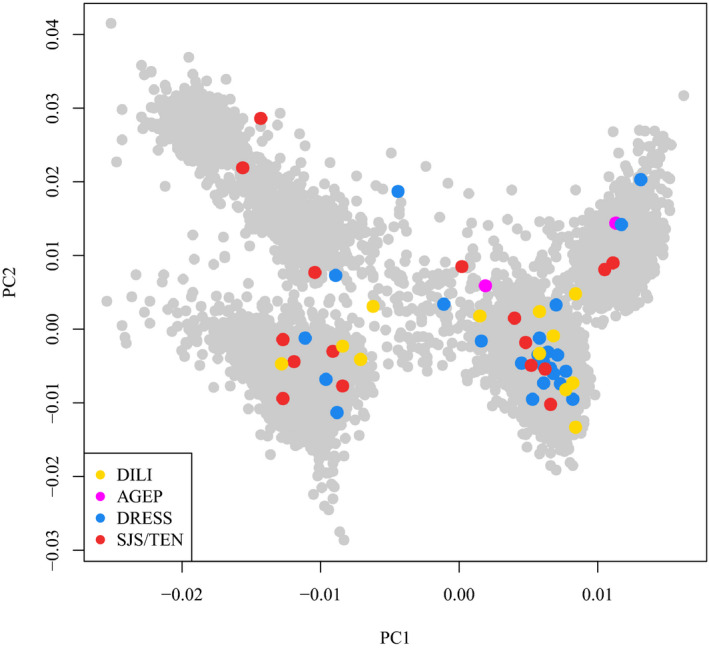
Scatterplot representing the first two principal components (PCs) of the current study cohort. Colored dots are the cases divided by clinical phenotypes and the gray dots are the controls. The controls cluster in four groups representing the Italian, Spanish, United Kingdom, and Swedish major control populations. AGEP, acute generalized exanthematous pustulosis; DILI, drug‐induced liver injury; DRESS, drug reaction with eosinophilia and systemic symptoms; SJS/TEN, Stevens‐Johnson syndrome/toxic epidermal necrolysis.

Our meta‐analysis included a total of 43 CBZ‐SCAR cases and 10,701 population controls from two genomewide association studies (GWAS) studies: British GWAS (14 cases and 2,263 controls) and broadly European GWAS (29 SCAR cases and 8,438 controls). The British GWAS included Northern European patients from the United Kingdom, which we have reported previously.^10^ The broadly European GWAS included a newly recruited set of CBZ‐SCAR cases with different clinical phenotypes (**Table** **1**) and European ancestries (**Figure S1**). The inferred population structure of the combined cohorts is shown in **Figure** **1**. A summary of the clinical characteristics of the SCAR cases is provided in **Table** **1**. We analyzed 25 patients with DRESS, 16 patients with SJS/TEN, and 2 patients with AGEP. The average age of patients at the time of adverse reaction was 34 years in both study phases. Nearly two‐thirds (63%) of cases were women.

**Table 1:** Clinical information of the CBZ‐SCAR and CBZ‐DILI cases included in the study^a^

Phenotype	Category	Phase I cases	Phase II cases
SCAR	Total number of cases	14	29
	Age at the onset in years (mean)	34	34
	Gender (% female)	43%	66%
	Allergy (% yes)	36%	24%
	Clinical subphenotypes		
	AGEP	1	1
	Hypersensitivity syndrome (DRESS)	13	12
	SJS	0	5
	SJS/TEN	0	10
	TEN	0	1
	Systemic symptoms		
	Eosinophilia	6	6
	Liver involvement	0	10
	Fever	13	22
	Pneumonitis	2	0
	Multi‐organ failure, death	1	0
	Additional evidences for the diagnosis		
	Skin biopsy	1	8
	Skin patch testing performed	1	5
	Multiple drug‐induced skin reactions	3	1
DILI	Total number of cases	0	12
	Age at the onset in years (mean)	–	37.6
	Gender (% female)	–	67%
	Pattern of liver injury		
	Cholestatic	–	3
	Mixed	–	2
	Hepatocellular	–	5
	Unknown	–	2
	Systemic symptoms		
	Eosinophilia	–	0
	Cutaneous rashes	–	0
	RUCAM score^b^		
	3–5 (possible)	–	2
	6–8 (probable)	–	6
	> 8 (highly probable)	–	2

AGEP, acute generalized exanthematous pustulosis; CBZ, carbamazepine; DILI, drug‐induced liver injury; DRESS, drug reaction with eosinophilia and systemic symptoms; RUCAM, Roussel Uclaf Causality Assessment Method; SCAR, serious cutaneous adverse reaction; SJS, Stevens‐Johnson syndrome; TEN, toxic epidermal necrolysis.

a Each patient can have more than one clinical characteristic; therefore, the numbers do not add up to the number of patients in phases I and II of the study.

b RUCAM scores were not possible for two patients recruited previously in the study of McCormack *et al*.^10^ because of the lack availability of all clinical information. Causality had been undertaken using temporal relationship to drug intake and exclusion of other causes.

Separately, we recruited 12 patients with CBZ‐DILI of European descent (**Figure** **1**). The type of liver injury in these patients was 50% hepatocellular and 50% cholestatic/mixed. None of them had cutaneous involvement or eosinophilia. Their clinical characteristics are also provided in **Table** **1**. CBZ‐DILI cases were compared with the 8,438 controls from the broadly European cohort.

### Meta‐analysis of CBZ‐SCAR

After quality control of the imputed data, we retained 5,271,349 single nucleotide polymorphisms (SNPs) for association analyses. Our meta‐analysis identified two loci attaining genomewide significance (*P *<* *5 × 10^−8^):

- The strongest association signal mapped to the major histocompatibility complex (MHC; lead SNP rs192543598; odds ratio (OR) = 18.1; 95% CI 8.03–40.90; *P *=* *1.7 × 10^−12^; P_perm_ **<* *5 × 10^−8^; **Table** **2** **,** **Table S1** **, Figure** **2** **, and** **Figure S2**), consistent with our previous result.^10^
  **Table 2:** Loci attaining genomewide significant evidence of association (*P* < 5 × 10^−8^) with CBZ hypersensitivity in a combined meta‐analysis of 43 patients and 10,701 controls of European ancestry

Locus	Lead SNP	Chr	Position	Alleles		Phase I		Phase II		Combined meta‐analysis				
				Risk	Other	OR (95% CI)	*P* value	OR (95% CI)	*P* value	OR (95% CI)	*P* value	Cochran's Q *P* value	Frequency in controls	Gnomad
MHC	rs192543598	6	29,931,345	G	A	127 (28.9–560)	1.5 × 10^−10^	7.8 (2.95–20.7)	3.5 × 10^−5^	18.1 (8.03–40.88)	1.7 × 10^−12^	0.002	0.01	0.02
*ALK*	rs187926838	2	29,818,291	G	A	15.3 (2.95–79.7)	0.001	11.0 (3.77–32.1)	1.1 × 10^−5^	12.1 (4.94–29.80)	4.9 × 10^−8^	0.74	0.007	0.008

CBZ, carbamazepine; Chr, chromosome; MHC, major histocompatibility complex; OR, odds ratio; SNP, single nucleotide polymorphism.
  **Figure 2:**
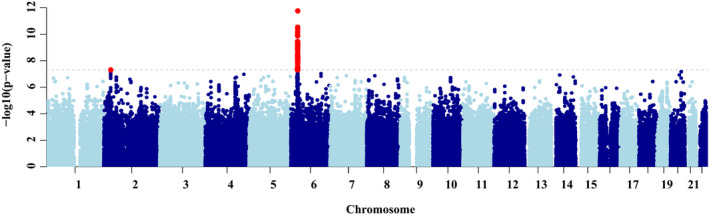
Manhattan plot displaying the results of the meta‐analysis of British and broadly European carbamazepine–serious cutaneous adverse reactions genomewide association study analyses. Single nucleotide polymorphisms in red have a significance level < 5 × 10^−8^.
- A novel association signal mapping to the *ALK* gene was observed outside the MHC (lead SNP rs187926838; OR = 12.1; 95% CI 4.94–29.80; *P *=* *4.9 × 10^−8^; *P*_perm_ = 1 × 10^−7^; **Table** **2** **and** **Figure S3**). The intronic lead SNP showed a consistent directional effect in both GWAS and was carried by 6% of the cases. The imputation for rs187926838 had high accuracy across the imputation batches (info score > 0.9). The frequency of rs187926838 in our control population was equal to the allele frequency reported in Europeans in gnomad (http://gnomad.broadinstitute.org/) and similar across platforms (**Table** **1** **and** **Table S1**), confirming the accuracy of the predicted genotypes. The rs187926838 showed a similar frequency in the SANAD study cohort,^2^ which has been exposed to a number of anti‐epileptic drugs, including CBZ (allele frequency = 0.005).

### Dissection of the MHC CBZ‐SCAR association signal

The lead SNP representing the MHC CBZ‐SCAR association signal, rs192543598, was in strong linkage disequilibrium (LD) with HLA‐A*31:01 (*r* ^2^ = 0.75). The HLA class I allele also reached genomewide significance in the meta‐analysis (OR = 8.0; 95% CI 4.10–15.80; *P *=* *2.2 × 10^−9^; P_perm_ **<* *5 × 10^−8^; **Table** **3** **and** **Table S2**). The phase II GWAS showed an MHC‐wide HLA‐A*31:01 association, replicating the previously reported signal.

**Table 3:** Imputed HLA alleles attaining nominal evidence of association (*P* < 0.01) with CBZ hypersensitivity in combined meta‐analysis of 43 patients and 10,701 controls of European ancestry

HLA allele	Phase I		Phase II		Combined meta‐analysis			
	OR (95% CI)	*P* value	OR (95% CI)	*P* value	OR (95% CI)	*P* value	Cochran's Q *P* value	Frequency in controls
HLA‐A*31:01	16.9 (5.40–52.40)	1.0 × 10^−6^	5.3 (2.29–12.40)	9.9 × 10^−5^	8.0 (4.10–15.80)	2.2 × 10^−9^	0.11	0.02
HLA‐B*51:01	2.8 (0.80–9.60)	0.10	3.7 (1.87–7.28)	0.0002	3.5 (1.91–6.27)	5.6 × 10^−5^	0.70	0.06
HLA‐C*15:02	2.1 (0.23–16.5)	0.47	4.1 (1.74–9.92)	0.001	3.7 (1.68–8.35)	0.002	0.56	0.02
HLA‐DPB1*09:01	4.9 (0.70–35.60)	0.12	4.5 (1.17–17.60)	0.03	4.6 (1.51–14.20)	0.007	0.96	0.009

CBZ, carbamazepine; OR, odds ratio.

The HLA‐A*31:01 association was less significant in the broadly European cases than in the British cases, probably because of greater heterogeneity in their geographic origin (**Figure** **1**). A lower allele frequency was observed in the Spanish cases (6%) compared with other ethnic groups (15% in Northern European and 13% Italian cases) despite an equivalent frequency in the three control groups. However, the effect of HLA‐A*31:01 was conserved in all European populations (OR_Italian_ = 8.7; OR_Spanish_ = 3.66; and OR_North Europeans_ = 5.51). There were no differences in allele frequencies of the GWAS variants in control groups despite the different genotyping arrays used.

Reciprocal conditional analyses demonstrated that rs192543598 and HLA‐A*31:01 represent the same underlying association signal (**Table S2**). After conditioning on the lead SNP, rs192543598, we observed some evidence for residual association (at locus‐wide significance, *P *<* *10^−5^) with CBZ‐SCAR, mapping to *MUC22* (lead SNP rs116071718, OR = 4.0; 95% CI 2.25–7.05; *P = *1.5 × 10^−6^; **Table S2** **and** **Figure S4**). After conditioning on both rs192543598 and rs116071718, the association signal with CBZ‐SCAR in the MHC was fully accounted for (**Figure S4**).

Marginal associations were also identified with HLA‐B*51:01 and HLA‐C*15:02 (**Table** **3**). These HLA alleles along with HLA‐A*31:01 constitute an uncommon haplotype present in European descent individuals (http://www.allelefrequencies.net). Joint carriage of the three alleles had a stronger signal compared with HLA‐A*31:01 alone (OR = 22.1, *P *=* *2.4 × 10^−6^ vs. OR = 5.7, *P *=* *6.6 × 10^−6^; **Table S3**).

### Assessment of polymorphic amino acid residues in HLA molecules

Predisposition to CBZ‐SCAR may be related to the same essential amino acid (AA) residues in different HLA alleles. Therefore, we performed an aggregated investigation by analyzing the polymorphic AA residues at these proteins. The strongest association was observed for isoleucine^73^ on the *HLA‐A* locus (OR = 5.5; 95% CI 2.94–10.40; *P *=* *1.4 × 10^−7^), but could not better explain the CBZ‐SCAR association than HLA‐A*31:01. The AA is shared by the A*33 and A*31 alleles.^16^ Conditional analysis to isoleucine^73^ or to HLA‐A*31:01 revealed that isoleucine^80^ in the B locus is a new independent factor, and it is shared with HLA‐B*57:01 among other HLA B alleles.^16^ After conditioning on both the sites, the residual independent association signal was still accounted for by rs116071718 in the *MUC22* gene (**Table S4**). These AA associations have not previously been found to be implicated in genetic predisposition to other adverse drug reactions.

### GWAS of CBZ‐SCAR clinical subtypes

We stratified the CBZ‐SCAR cases by clinical phenotypes (AGEP, DRESS, and SJS/TEN; see Methods). The AGEP analysis was not performed because we had only two cases, although it is worth noting that one of the two cases was positive for HLA‐A*31:01.

In the CBZ‐DRESS subgroup, HLA‐A*31:01 and its proxy SNP, rs192543598, reached genomewide significance (*P*_HLA‐A*31:01_ = 2.1 × 10^−9^ and *P*_rs192543598_ = 2.4 × 10^−13^). Consistent with the literature,^17^ HLA‐A*31:01 showed a stronger effect with CBZ‐DRESS than with SJS/TEN (OR = 12 vs. OR = 2.5, respectively; **Table** **4**) with no significant association in the latter group. There was no other phenotype‐specific genomewide significant association for both DRESS and SJS‐TEN (**Figure S5**).

**Table 4:** Association effect size of HLA‐A*31:01 across different clinical phenotypes

Clinical groups	*N*	OR (95% CI)	*P* value	Allele frequency in cases	Allele frequency in controls
SJS/TEN	16	2.4 (0.55–10.59)	0.2	0.06	0.02
DRESS	25	12.9 (5.58–29.78)	2.1 × 10^−9^	0.14	0.02
DILI	12	7.3 (2.47–23.67)	0.0004	0.17	0.02
All clinical phenotypes (SCAR and DILI)	55	8.2 (4.56–14.66)	1.8 × 10^−12^	0.14	0.02

DILI, drug‐induced liver injury; DRESS, drug reaction with eosinophilia and systemic symptoms; OR, odds ratio; SCAR, serious cutaneous adverse reaction; SJS, Stevens‐Johnson syndrome; TEN, toxic epidermal necrolysis.

The effect of rs187926838 in the *ALK* gene was conserved among the CBZ‐SCAR clinical subtypes (**Table S5**). We also found that the SJS/TEN phenotype showed an MHC‐region‐wide significant association with HLA‐B*57:01 (OR = 6.2; 95% CI 2.47–15.37; *P *=* *9.9 × 10^−5^). HLA‐B*57:01 was in strong LD (*r* ^2^ = 0.8) with the most highly associated SNP in the MHC region (rs116347890; OR = 11.0; 95% CI 4.62–26.42; *P *=* *6.6 × 10^−8^) but was independent of HLA‐A*31:01 (OR = 6.4; 95% CI 2.55–16.11; *P *=* *7.6 × 10^−5^) after conditioning on HLA‐A*31:01. The polymorphic AA position 97 showed an association when valine was present (OR = 6.0; 95% CI 2.45–15.18; *P *=* *0.0001). Valine^97^ is specific for HLA‐B*57:01, HLA‐B*57:02, and HLA‐B*57:03 and other very rare B57 alleles.^16^

### GWAS of CBZ‐DILI

GWAS analysis of 12 European CBZ‐DILI cases against 8,438 European controls did not identify a genomewide association because of limited power. However, 33% of cases were HLA‐A*31:01 carriers (OR = 7.3; 95% CI 2.47–23.67; *P *=* *0.0004), although the OR was less than that seen with DRESS (OR = 12.9). Our prediction of the association with HLA‐A*31:01 was fully confirmed by HLA sequencing in 11 cases (DNA was no longer available in one patient). CBZ‐DILI cases did not show an association with rs187926838 (in the *ALK* gene), distinct from that seen with CBZ‐SCAR cases (**Table S5**). The most significant AA residues associated with CBZ‐DILI were isoleucine^73^ (OR = 7.29; 95% CI 2.578–20.6; *P *=* *0.0002) and threonine^9^ (OR = 5.5; 95% CI 2.31–13.14; *P *=* *0.0001) at the *HLA‐A* locus. The two residues were in LD (*P*_73I conditional to 9T_ = 0.2). Moreover, there was enrichment of HLA‐A*31:01 carriers in the 10 patients with CBZ‐SCAR with liver involvement compared with the remaining cases (25% vs. 12%, respectively).

Taking all the cases of CBZ‐induced hypersensitivity reactions (SCAR and DILI) together, the risk of developing either DILI or SCAR when given CBZ was eightfold higher in cases carrying HLA‐A*31:01 (OR = 8.2; 95% CI 4.56–14.66; *P = *1.8 × 10^−12^; **Table** **4**).

## Discussion

In the current study, we show that HLA‐A*31:01 is the strongest predictor of CBZ‐SCAR in a European‐ancestry population, extending our previous study in Northern Europeans^10^ to include Southern Europeans. In addition, we also show that (i) HLA‐A*31:01 seems to predispose to CBZ‐induced liver injury, and (ii) a variant in the *ALK* gene was associated with an increased risk of CBZ‐SCAR but not CBZ‐DILI.

Our finding of the association between CBZ‐SCAR and HLA‐A*31:01, initially reported in 2011,^10^ has also been reported by Amstutz *et al*.^18^ in a multiethnic North American pediatric cohort and by Genin *et al*.^17^ in a European SCAR cohort. In our study, HLA‐A*31:01 was a stronger risk factor for DRESS than SJS/TEN, in line with Genin *et al*.^17^ (OR = 57.6 vs. OR = 4.4, respectively) and Amstutz *et al*.^18^ (OR = 31.5 vs. OR = 2.8, respectively), but not with our original study (OR = 12 vs. OR = 25, respectively)^10^ where numbers were much smaller. It is likely that HLA‐A*31:01 is the most important shared risk factor across CBZ hypersensitivity phenotypes, with the strongest predisposition being to DRESS. Indeed, our meta‐analysis shows that HLA‐A*31:01 carriers have eightfold higher risk of developing CBZ‐SCAR than noncarriers. Interestingly, a recent prospective study in Japan was able to show that pre‐prescription genotyping for HLA‐A*31:01 reduces the incidence of CBZ hypersensitivity reactions.^19^ Taken together, the overwhelming evidence of the role of HLA‐A*31:01 in predisposing to CBZ‐SCAR shows that there is a need to implement its use in clinical settings, as outlined in the recent Clinical Pharmacogenetics Implementation Consortium guideline.^20^ Independent of the HLA‐A*31:01 association, we identified a residual effect in the MHC region mapping to *MUC22* gene (lead SNP rs116071718). *MUC22* codes for panbronchiolitis‐related mucin‐like protein 1 and has been associated with SJS/TEN caused by a variety of drugs, including CBZ in European patients.^21^ Its role in SCAR needs further investigation. We also found that HLA‐B*51:01 was the second most significant HLA allele. HLA‐B*51:01 has already been reported to be marginally associated with CBZ‐SCAR^12^, ^13^, ^14^ and more recently with SCAR due to other drugs.^22^ HLA‐B*51:01 together with HLA‐A*31:01 and HLA‐C*15:02 constitute a haplotype that has a larger OR than each single allele (**Table S3**). This is an interesting finding, which suggests that *in vivo*, the T‐cell response to CBZ‐derived antigens requires cooperativity between different HLA alleles, as we have previously demonstrated in an HLA‐A*31:01‐positive patient.^23^

We also identified an association between CBZ‐induced SJS/TEN and HLA‐B*57:01. This allele is a well‐known risk factor for other CD8+ T‐cell‐mediated reactions, including abacavir hypersensitivity syndrome^24^, ^25^ and flucloxacillin‐induced DILI,^26^ and more recently, DILI induced by two other drug combinations, pazopanib and a combination of antituberculosis and anti‐HIV drugs.^27^, ^28^ Given that the association with CBZ‐induced SJS/TEN was not genomewide significant, it needs replication in other cohorts. Interestingly, our finding of the association of valine^97^ with CBZ‐SJS/TEN is in line with the association of valine^97^ with flucloxacillin‐DILI,^29^ suggesting that the binding site of HLA‐B*57:01 may be promiscuous for a number of drugs, which would be in keeping with the increasing number of reports of immune‐mediated reactions associated with this allele.

We have, we believe, for the first time evaluated whether HLA‐A*31:01 is a risk factor for DILI. Taking all cases of DILI into account, the carriage rate of HLA‐A*31:01 was 33% with an OR of 7, higher than that found in association of HLA class II alleles with lumiracoxib‐induced DILI.^30^ Interestingly, CBZ‐SCAR and CBZ‐DILI also shared the most significant AA, isoleucine.^73^ Isoleucine^73^ is a cryptic epitope specific for A31 and A33 antigens.^31^ Position 73 is not normally exposed. When the antigen changes its conformation and β2m and peptide dissociate from the heavy chain, isoleucine^73^ is externalized with the potential to lead to an autoimmune reaction.^32^ Interestingly, HLA‐A*33:01 and HLA‐A*33:03 have recently been associated with DILI due to several unrelated drugs.^33^ The association with HLA‐A*31:01, however, was not genomewide significant, which may reflect the small numbers studied. It may also reflect that the mechanism of antigen presentation (be it the parent drug or metabolite) differs between the skin and liver given the major role of the liver in drug metabolism and its ability to form chemically reactive intermediates.^34^ Further work in larger numbers of patients with CBZ‐DILI, together with mechanistic studies, will be needed to understand the role of drug/metabolites as antigens in the context of different drug metabolizing and antigen presentation capabilities, and, indeed, whether these differences are responsible for the remarkable organ‐specificity of the reactions and their severity seen in different patients.

A tantalizing association that we have identified in our meta‐analysis (that did not pass genomewide significance after permutation) that was observed in the SCAR but not in the DILI cases was with uncommon variants in the *ALK* gene, which codes for the anaplastic lymphoma kinase gene. Somatic mutations in the *ALK* gene have been identified in different cancers,^35^ including lung cancer, which has led to the development of ALK‐inhibitors for therapy. The product of the *ALK* gene, a receptor tyrosine kinase belonging to the insulin receptor family, seems to be important for the balance between proliferation and apoptosis.^36^ Furthermore, the associated region falls within a keratinocyte‐specific predicted insulator (**Figure S6**). Given that ALK is important in cellular proliferation and cell death and shows ubiquitous tissue distribution (http://www.proteinatlas.org/ENSG00000171094-ALK/tissue), it may have an important role in T‐cell proliferation and keratinocyte death, both important in the pathogenesis of SCAR.

In conclusion, we have provided further data regarding genetic factors predisposing to different CBZ adverse reaction phenotypes, which are thought to have an immune pathogenesis, namely SCAR and DILI. We have extended our studies to include Southern Europeans in addition to our previous study in Northern Europeans^10^ and included an analysis of CBZ‐DILI. We have also identified novel associations with the *ALK* and *MUC22* genes, which require further validation and experimental investigation to determine the mechanisms. It is possible that there are also other genetic factors outside the MHC region, but we may not have had the statistical power to detect them in this study. It is, therefore, important that further work also involves a trans‐ethnic meta‐analysis of the GWAS in diverse populations, which have been undertaken to date, to fully assess differences in the genetic architecture of CBZ adverse reactions between ancestries and, more precisely, localize the underlying causal alleles.

## Methods

### Study design overview

Our study combines results from two GWAS conducted in subjects with ancestry from Northern Europe, Italy, and Spain. All participants provided written informed consent and each study was approved by the appropriate national or institutional ethical review boards. Because the reactions have a very low prevalence, we used general population samples as study controls.

### British CBZ‐SCAR GWAS

The study included 14 patients with CBZ‐hypersensitivity of Northern European descent of the 21 recruited at the University of Liverpool, as described by McCormack *et al*.^10^ A total of 2,263 population controls of Northern European descent from the Wellcome Trust Case Control Consortium were utilized. The other Liverpool samples (*n* = 7) originally described were excluded either because of population stratification issues or because two patients had DILI rather than skin involvement (and so have been included in the liver analysis).

### Broadly European CBZ‐SCAR GWAS

The study included a total of 29 CBZ‐SCAR samples obtained from two available sources: 3 cases were from the PGX40001 study^37^ and 26 were newly recruited between 2009 and 2013 as part of the International Consortium of Drug Hypersensitivity (ITCH) study. The ITCH study was run under the auspices of the International Serious Adverse Event Consortium, involving 12 recruitment centers in Europe, Australia, and South America. Clinical inclusion criteria for all ITCH‐related SCAR cases were as described by our previous phenotype standardization protocol for drug‐induced skin injury.^38^ All patient phenotypes were independently adjudicated by the co‐authors N.S. and P.F. Further clinical details are provided in **Table** **1**.

For this group, a total of 8,438 population controls of European descent were selected from the Wellcome Trust Case Control Consortium (not overlapping with samples from the British cohort), the population reference sample (POPRES),^39^ the PGX40001 study,^37^ the Spanish Bladder Cohort (dbGaP phs000346.v1),^40^ the Hypergenes cohort (http://www.hypergenes.eu), the National Spanish DNA Bank cohort (http://www.bancoadn.org/), the Swedish Twin Registry cohort (http://ki.se/en/research/the-swedish-twin-registry), LAM30004 study,^37^ TSI (Hapmap data), and the iSAEC Italian Penicillin Tolerant Cohort (IPTC).

### Broadly European CBZ‐DILI GWAS

As part of the phase II study, we also collected 12 patients of European descent with CBZ‐DILI, in which the liver was involved by itself. The cases were recruited from two large international DILI consortia: iDILIC and DILIN,^33^ whereas two other cases were as described previously.^10^ The iDILIC cases were evaluated by application of the Council for International Organizations of Medical Science (CIOMS) scale, also called the Roussel Uclaf Causality Assessment Method (RUCAM), and by expert review by a panel of three hepatologists. Only cases having at least possible causality (score ≥ 3) were included in the study.^33^ DILIN causality assessment is determined by a panel of three hepatologists who independently assign a causality score ranging from 1 (definite) to 5 (unlikely) as well as a severity score ranging from 1 (mild) to 5 (fatal), as previously described.^33^ Besides the DILI phenotype, DILIN and iDILIC also collected additional clinical information, including the occurrence of eosinophilia and cutaneous involvement. The cases were compared with the 8,438 phase II population controls of European descent.

### Genotyping of cases and controls

Out of the cumulative 43 CBZ‐SCAR cases, three PGX40001 cases were previously genotyped with Illumina 1M Duo chip.^41^ DNA from the rest of British and broadly European cases was extracted from whole blood and stored in the Wolfson Centre for Personalized Medicine in Liverpool. Genomewide genotyping was profiled by the Illumina Infinium HumanCoreExome Bead Chip for 16 cases and by Illumina HumanOmniExpress Bead Chip for 24 cases at the Broad Institute, Boston. Among the CBZ‐DILI cases, three cases were previously genotyped with Illumina 1M Duo Chip, whereas two cases were newly profiled by HumanCoreExome Bead Chip and seven cases by HumanOmniExpress Bead Chip at the Broad Institute, Boston.^33^ A total of 10,701 previously genotyped population controls were cumulatively used in British and broadly European cohorts. Information about the genotyping platform used by each of the control cohorts is reported in **Table S6**. For each of the sample batches (defined as a set of subjects—either cases or controls—genotyped together by the same array), quality control was conducted at both SNP and subject levels before performing the imputation as previously described.^41^ Analysis of population structure was performed by the EIGENSTRAT package version 3.0.^42^ Pre‐phasing and imputation were performed in batches by dividing the cases and controls according to genotyping platform, using SHAPEIT (version v2.r727)^43^ and IMPUTE2 (version 3)^44^ with 1000 Genomes Project (release version 3) as reference.^43^ For downstream analysis, we used best‐guess genotypes retaining imputed genotypes with posterior probability > 0.9. Detailed methods are outlined in the **Supplementary Materials**.

### Association analysis and meta‐analysis

We tested for association of each SNP with CBZ‐SCAR, separately in British and broadly European GWAS, in a logistic regression framework, under an additive genetic model, with adjustment for the principal components from smartPCA to account for population structure using PLINK version 1.07.^45^ No other additional covariates were included in the model because we did not have clinical information for controls. Association summary statistics from the two phases were combined using effective sample size weighted *z*‐score fixed‐effects meta‐analysis, implemented in METAL.^46^ Allelic ORs across the two phases were obtained through inverse‐variance weighting of effect sizes, with heterogeneity assessed with Cochran's *Q* statistic,^47^ implemented in METAL. We reported only those SNPs that attained, in addition to genomewide significance, nominal evidence of association (*P *<* *0.05) with the same direction of effect on CBZ‐SCAR in both GWAS phases (internal validation). Furthermore, we tested for association of each SNP with CBZ‐SCAR clinical subtypes: SJS/TEN in phase II GWAS and DRESS across both phases in the same meta‐analysis framework. We also tested for association of each SNP with CBZ‐DILI in phase II GWAS, in a logistic regression framework, under an additive genetic model, with adjustment for the principal components to account for population structure. Genomewide significance was defined using a common threshold of *P *<* *5 × 10^−8^. To account for the small sample size and the disproportionate case/control ratio, we applied a permutation approach for genomewide significant signals. In particular, we randomly permuted genotypes among individuals within the same phase of the design, tested for association, and then meta‐analyzed. We applied 5 × 10^8^ permutations to demonstrate genomewide significance. All detailed analyses and Manhattan plots were performed with R version 3.0.2.^14^

### HLA imputation, genotyping, and analysis

For each batch, HLA alleles were inferred using HLA genotype imputation with attribute bagging^48^ using the reference predictor panels specific for the genotyping chip. AA changes were inferred by SNP2HLA using reference data collected by the Type 1 Diabetes Genetics Consortium.^49^ We tested for association of carriage of each allele/AA/specific HLA haplotypes with CBZ‐SCAR, CBZ‐SCAR subtypes, and CBZ‐DILI using the same methods described above. MHC significance was defined using the Bonferroni correction threshold of *P* < 0.00025 accounting for 200 observed HLA alleles across loci (0.05/200). Conditional analyses in the MHC region were undertaken and the genotypes at the conditioning SNP(s) were included as covariates under an additive model. Fixed‐effects meta‐analyses across the two phases of GWAS were performed using the methods described above for unconditional analyses. High‐resolution genotyping of HLA loci was performed on all DILI cases by Histogenetics (Ossining, NY), as previously described.^33^

## Funding

This work was supported by the International Serious Adverse Events Consortium (iSAEC). The iSAEC is a nonprofit organization dedicated to identifying and validating DNA‐variants useful in predicting the risk of drug‐related serious adverse events. The Consortium brings together the pharmaceutical industry, regulatory authorities, and academic centers to address clinical and scientific issues associated with the genetics of drug‐related serious adverse events. The iSAEC's current funding members include: Abbott, Amgen, AstraZeneca, Daiichi Sankyo, GlaxoSmithKline, Merck, Novartis, Pfizer, Takeda, and the Wellcome Trust. M.P. is an NIHR Senior Investigator. M.P. and A.A. thank the MRC Centre for Drug Safety Science for support (MR/L006758/1). P.N. was supported by iSAEC. A.P.M. is a Wellcome Trust Senior Fellow in Basic Biomedical Science (under award WT098017). This is a summary of independent research partly (the DILIGEN and iDILIC sample collection) funded by the National Institute for Health Research (NIHR) Nottingham Digestive Diseases Biomedical Research Unit at the Nottingham University Hospitals NHS Trust and University of Nottingham. The DILIN (https://dilin.org/) is supported by the National Institute of Diabetes and Digestive and Kidney Diseases of the National Institutes of Health (NIH) as a Cooperative Agreement (U01s). The Spanish DILI Registry is partly funded by the Spanish Medicine Agency, Fondo Europeo de Desarrollo Regional (FEDER; FIS 12/00378, FIS 16/01748). CIBERehd is funded by Instituto de Salud Carlos III. The Swedish case collection SWEDEGENE (www.swedegene.se) has received support from the Swedish Medical Products Agency, the Swedish Society of Medicine (2008‐21619), Swedish Research Council (Medicine 521‐2011‐2440 and 521‐2014‐3370), and Swedish Heart and Lung Foundation (20120557 and 20140291). The EUDRAGENE collaboration received support from the EC 5th Framework program (QLRI‐CT‐2002‐02757). M.M. is supported by the National Institute for Health Research (NIHR) Biomedical Research Centre at Guy's and St. Thomas’ NHS Foundation Trust and King's College London. The views expressed are those of the author(s) and not necessarily those of the NHS, the NIHR, or the Department of Health.

## Conflict of Interest

P.N. is an employee of Sema4 Mount Sinai venture, Stamford, CT, USA. M.R.N. is an employee of GlaxoSmithKline. All other authors declared no competing interests for this work.

## Author Contributions

P.N., A.K.D., M.R.N., G.A., A.P.M., A.A., and M.P. wrote the manuscript. P.N., A.P.M., M.R.N., M.J.D., A.K.D., G.A., Y.S., A.A., and M.P. designed the research. P.N., A.S., and A.P.M. analyzed the data. M.P., A.K.D., P.B.W., S.B., L.M., G.A., M.I.L., R.J.A., M.W., P.H., C.S., E.S.B., P.F., K.K., T.L., A.M., M.M., E.P., W.P., A.R., N.S., G.S., L.K.T., and A.F. contributed new reagents/analytical tools.

## Supporting information


**Supplementary Methods, Figures, and Tables.**
