# Supplementary material for: Shared Genetic Risk Factors Across Carbamazepine‐Induced Hypersensitivity Reactions
Source: Clin Pharmacol Ther. 2019 Jul 3;106(5):1028–36. doi: 10.1002/cpt.1493 (PMC7156285; doi:10.1002/cpt.1493)
Supplement: Supplementary file 1 — Supplementary Methods, Figures, and Tables. [file CPT-106-1028-s001.pdf]

Shared genetic risk factors across Carbamazepine-induced adverse reactions

Paola Nicoletti MD, PhD[1,2] Sarah Barrett BSc[3], Laurence McEvoy BSc [3], Ann K Daly PhD[4], Guruprasad Aithal MD, PhD[5], M. Isabel Lucena MD, PhD [6], Raul J Andrade MD, PhD [6], Mia Wadelius MD[7], Pär Halberg MD PhD[7], Camilla Stephens PhD[6], Einar S Bjornsson MD, PhD[8], Peter Friedman[9] FRCP, Kati Kainu[10] MD,PhD, Tarja Laitnen[10] MD, PhD, Anthony Marson MD, FRCP [3], Mariam Molokhia PhD[11], Elizabeth Philips PhD[12], Werner Pichler MD[13], Antonino Romano MD[14], Neil Shear[15] MD, Graeme Sills[3] PhD, Luciana K. Tanno MD PhD[16], Ashley Swale PhD[17], Aris Floratos PhD[17], Yufeng Shen PhD[17], Matthew R. Nelson PhD[18], Paul B. Watkins MD [19], Mark J Daly PhD [20], Andrew P Morris PhD[2,21], Ana Alfirevic[3] MD PhD, Munir Pirmohamed[3] FRCP, PhD

Table of content

Supplementary Methods……………………………………………………………2

Supplementary Figures…………………………………………………………….3

Supplementary Tables……………………………………………………………..9

Supplementary Methods

Population structure

To assess the extent of population structure of the two phases, and derive eigenvectors to account for confounding, we applied principal component analysis on British and broadly-European cohorts separately, using the smartPCA program from the EIGENSTRAT package (version 3.0)(1) on the overlapping SNPs (minor allele frequency, MAF>0.01) across the range of genotyping arrays used for typing cases and controls. We used the first twelve and seven components as covariates in the statistical model respectively for British and broadly-European analysis.

Pre-phasing and imputation

Pre-phasing and imputation was performed in batches by dividing the cases and controls according to genotyping platform. For each batch, we first pre-phased the genotype scaffold using SHAPEIT (version v2.r727)(2). Imputation was then undertaken using IMPUTE2 (version 3)(3) using the reference panel from the 1000 Genomes Project (release v3)(2). We used the "all ancestries" reference panel to improve the quality of the imputation especially of lower frequency variants(3). For downsteam analysis, we used best-guess genotypes retaining imputed genotypes with posterior probability > 0.9. In the combined dataset, we then retained SNPs with: (a) no significant difference in missingness between cases and controls (P>0.0001); (b) no significant deviation from Hardy-Weinberg equilibrium (P>0.0001); (c) less than 5% missing genotypes in all batches; (d) imputation info score greater than 0.8 in all batches; and (e) minor allele frequency (MAF) in the European descent haplotypes from the 1000 Genomes Project reference panel of at least 0.01. Finally, we compared SNP allele frequencies in controls between genotyping platforms. Specifically, we tested for association of genotyping platforms with SNPs in same ethnicity controls in a logistic regression framework, under an additive genetic model, with adjustment for principal components from smartPCA to account for population structure. SNPs with nominal evidence of association (P<0.005) were excluded. Sex chromosomes and mitochondria were not imputed.

Supplementary Figures

Figure S1: Scatterplot representing the first two principal components of the current study cohort. Red dots are the phase I (14 cases vs 2263 controls) and the gray dots are phase II cohorts (41 cases –SCAR and DILI- and 8,438 controls). The phase I cohort included only UK samples.

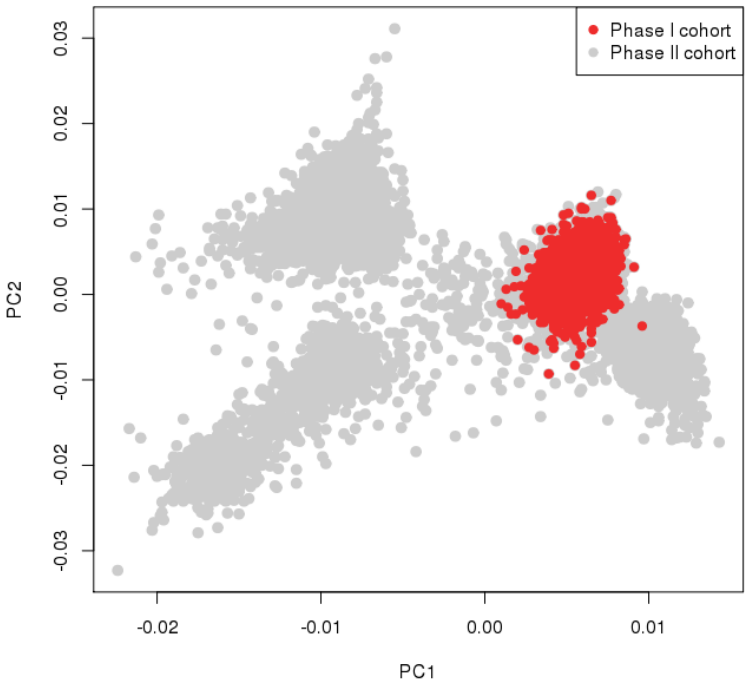

Figure S2 QQ plots for the meta-analysis (Gnomic Inflation factor = 1.01)

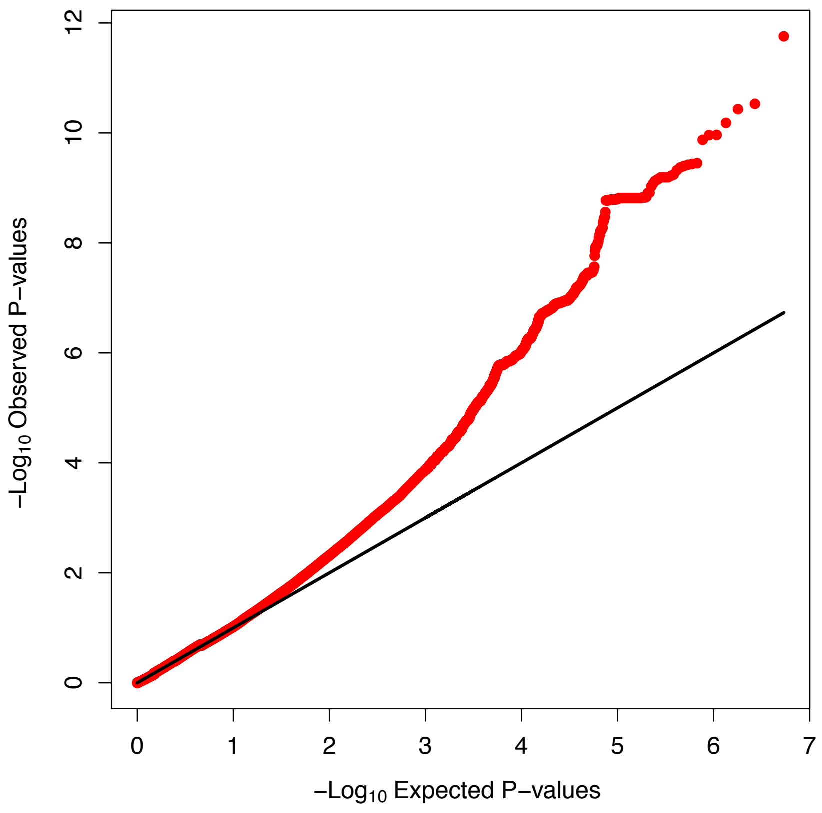

Figure S3: Local Manhattan plot of meta-analysis results generated using LocusZoom. Shown is rs187926838 and flanking region. Regional plots were drawn by LocusZoom(4).

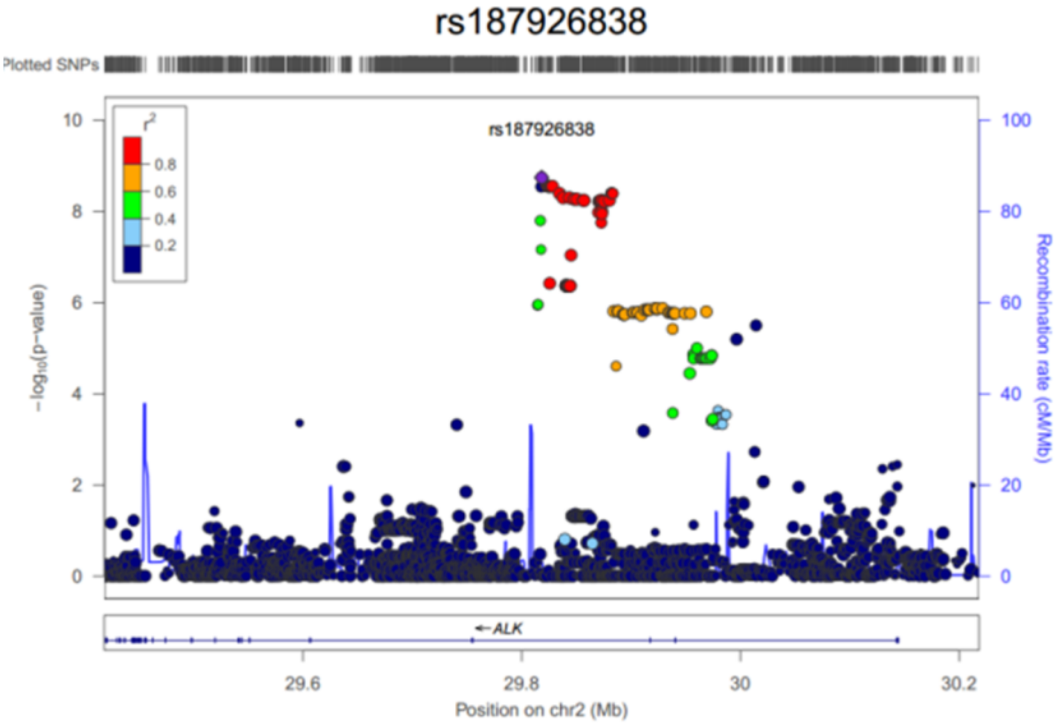

Figure S4 Manhattan plots displaying the results within 500kb across rs192543598 locus of original (A) and conditioned to A*33:01 (B), conditioned to rs192543598 and rs116071718 (C).

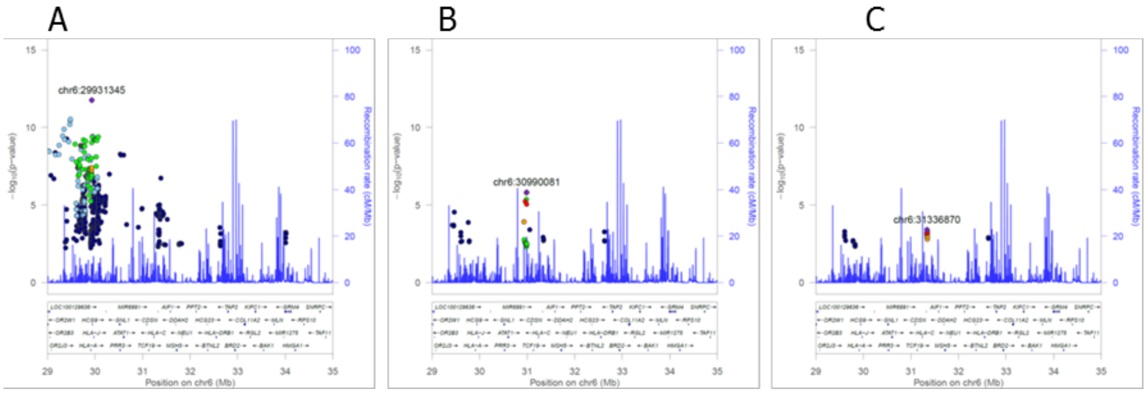

Figure S5: Manhattan plot displaying the results of the clinical phenotypes: (A) DRESS meta-analysis, the results are plotted if they have been filtered to have p<0.05 in both phases and association with the same direction of effect; (B) SJS/TEN GWAS analysis; (3) DILI GWAS analysis. SNPs with association p-value > 0.02 were not plotted.

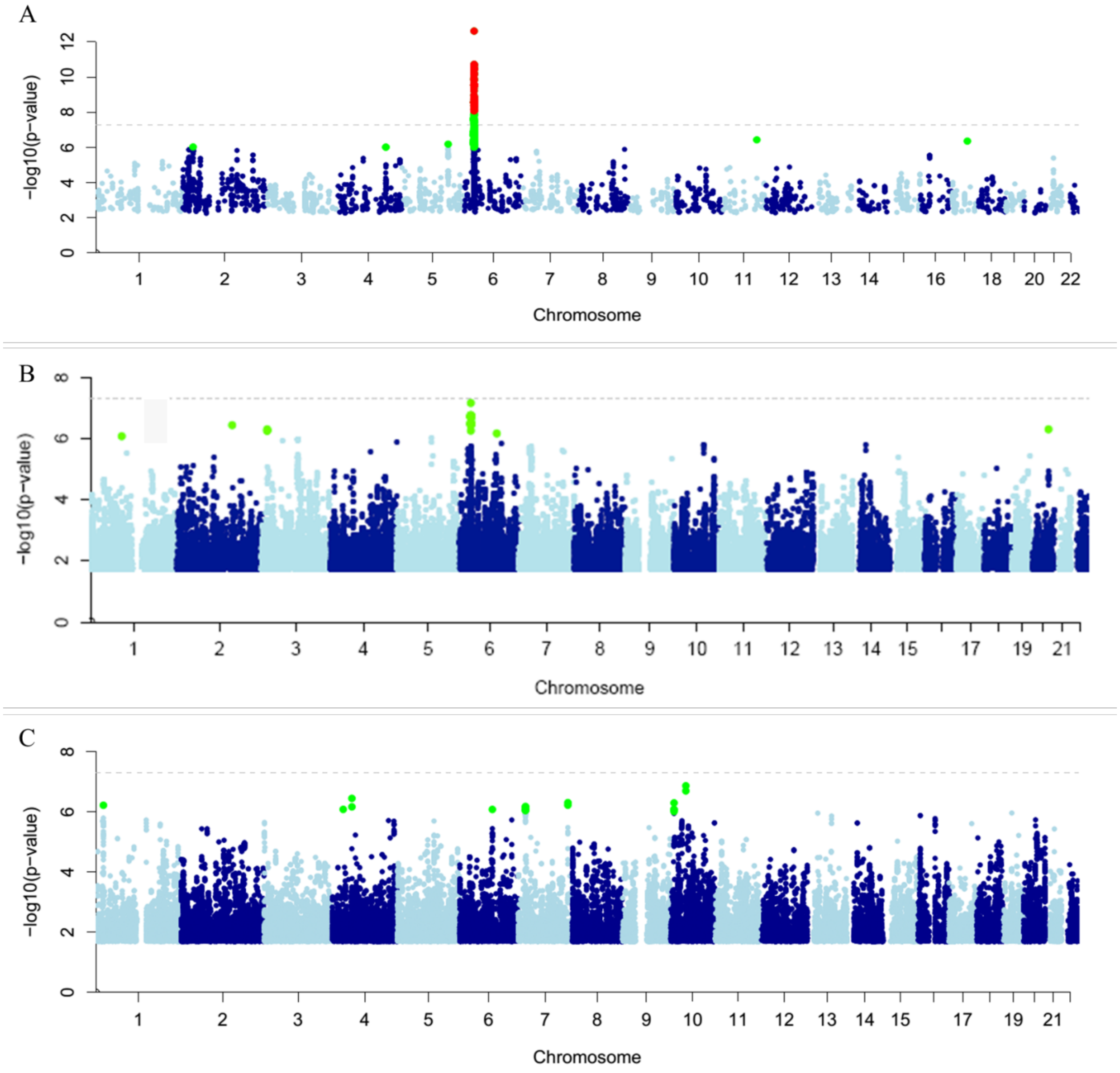

Figure S6: Genome browser screenshot of the region surrounding the top associated SNP on chromosome 2**.**

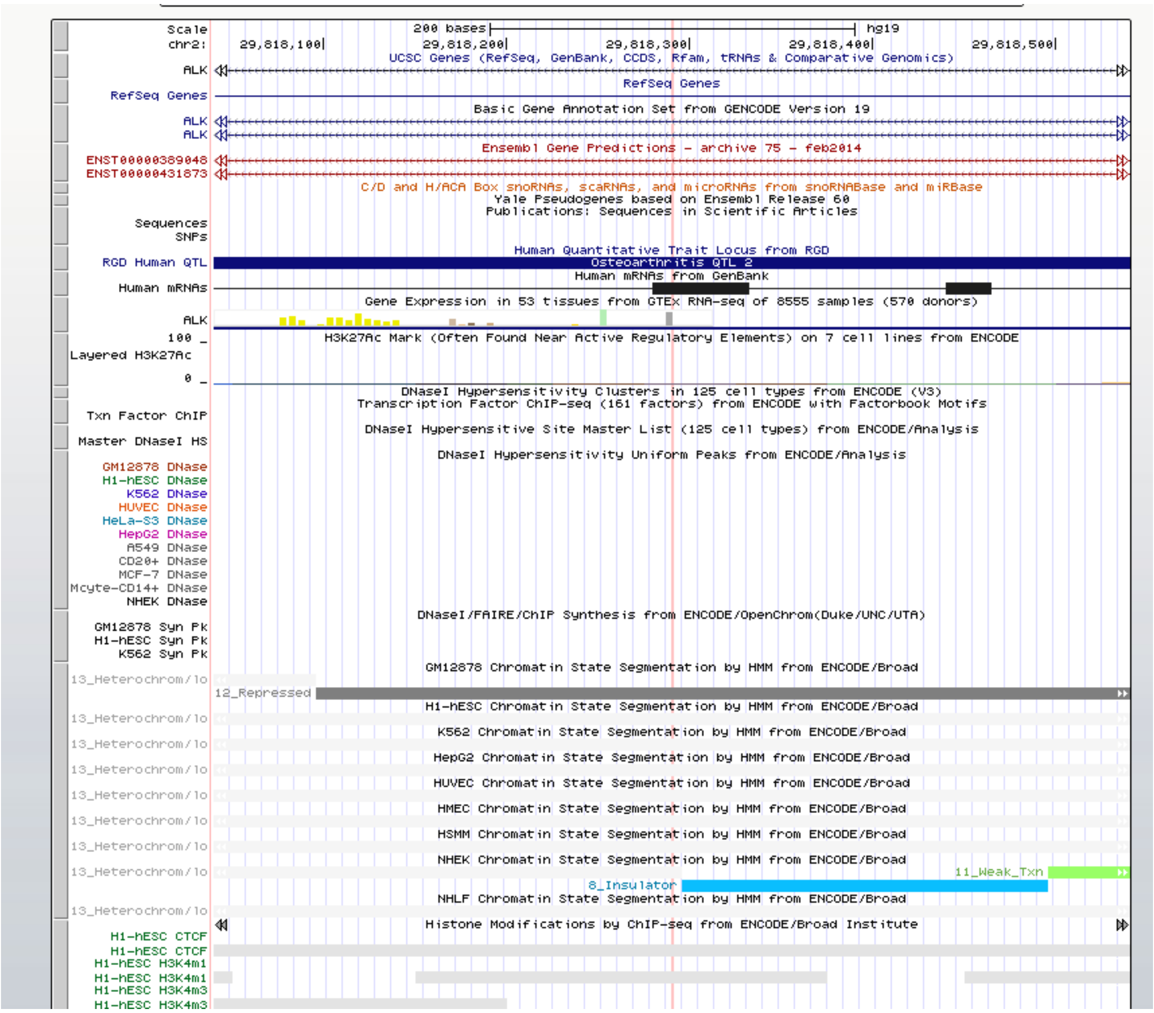

Supplementary Tables

Table S1: Allele frequency of the genome-wide significant loci in controls considering different European groups and genotyping arrays.

Markers	Caucasians			Spanishs		Italians		Noeth Europeans
	Illumina 1 M	HumanOmniExpress	HumanCoreExome	Illumina 1 M	HumanCoreExome	Illumina 1 M	HumanOmniExpress	Illumina 1 M
A*31:01	0.03	0.03	0.04	0.03	0.04	0.02	0.03	0.03
rs192543598	0.01	0.02	0.02	0.01	0.02	0.02	0.03	0.01
rs187926838	0.005	0.01	0.008	0.005	0.008	0.007	0.01	0.005

Table S2: Effect size of the association of the top associated variants within MHC region before and after conditional analysis on A*31:01 and rs192543598

	Original				COND A*31:01				COND rs192543598			
**Cohort**	**OR**	**LCI**	**UCI**	**PV**	**OR**	**LCI**	**UCI**	**PV**	**OR**	**LCI**	**UCI**	**PV**
**PHASE I**												
**A*31:01**	16.88	5.44	52.41	1.012E-06	-	-	-	-	N/A	N/A	N/A	1.00
**B*51:01**	2.81	0.82	9.58	0.10	1.92	0.52	7.14	0.33	0.63	0.12	3.40	0.59
**B*57:01**	-	-	-	-	-	-	-	-	-	-	-	-
**rs116071718**	3.44	1.27	9.29	0.01	4.00	1.41	11.29	0.01	6.21	1.93	19.99	0.002
**rs192543598**	127.30	28.91	560.40	1.47E-10	N/A	N/A	N/A	0.99	-	-	-	-
**PHASE II**												
**A*31:01**	5.32	2.29	12.36	0.0001	-	-	-	-	5.69	0.77	41.88	0.09
**B*51:01**	3.69	1.87	7.28	0.0002	3.06	1.53	6.10	0.002	2.68	1.30	5.52	0.007
**B*57:01**	3.56	1.59	7.94	0.002	4.05	1.79	9.13	0.0008	4.06	1.79	9.23	0.0008
**rs116071718**	3.29	1.72	6.28	0.0003	3.25	1.68	6.28	0.0005	3.47	1.80	6.67	0.0002
**rs192543598**	7.81	2.95	20.68	0.00003	1.42	0.16	12.68	0.7553	-	-	-	-
**META-ANALISYS**												
**A*31:01**	8.03	4.09	15.8	2.20E-09	-	-	-	-	5.69	0.77	41.88	0.99
**B*51:01**	3.46	1.91	6.27	0.000056	1.72	0.66	4.51	0.002	2.14	1.10	4.16	0.06
**B*57:01**	3.56	1.59	7.94	0.002	4.05	1.79	9.13	0.0008	4.06	1.79	9.23	0.0008
**rs116071718**	3.32	1.92	5.74	0.00002	3.45	1.97	6.01	0.00001	3.99	2.25	7.05	0.0000015
**rs192543598**	18.11	8.03	40.88	1.70E-12	1.42	0.16	12.68	0.797	-	-	-	-

OR = Odd Ratio; ULC= 95% lower Confidence interval of the Odd Ratio; ULC= 95% upper Confidence Interval of the Odd Ratio; PV = logistic p-value

Table S3: Effect size of the association of the different haplotype involving HLA-A*31:01, HLA-B*51:01 and HLA-C*15:02 HLA alleles

HLA-A*31:01|HLA-B*51:01|HLA-C*15:02	phase1				phase 2				Meta-analysis			
	MAF_A	MAF_UN	OR	P	MAF_A	MAF_UN	OR	P	MAF_A	MAF_UN	OR	P
**PPP**	0.03	0.002	**19.40**	0.046	0.05	0.004	**23.10**	1.35E-05	0.04	0.003	**22.10**	2.46E-06
**APP**	0.004	0.01	**0.39**	0.8	0.06	0.02	**3.09**	0.05	0.04	0.02	**2.35**	0.15
**AAP**	0	0.003	**-**	-	-	-	-	-	0.00	0.003	-	-
**PPA**	0.02	0.001	**140.00**	0.01	-	-	-	-	0.01	0.00	**11.60**	0.1
**APA**	0.05	0.02	**1.96**	0.5	0.09	0.04	**2.60**	0.05	0.07	0.04	**2.46**	0.04
**PAA**	0.16	0.02	**14.10**	0.00003	0.075	0.02	**3.88**	0.01	0.10	0.02	**5.72**	6.61E-06
**AAA**	0.73	0.94	**0.17**	0.0002	0.735	0.91	**0.25**	0.00001	0.74	0.92	**0.22**	1.03E-08

The order of the HLA alleles in the haplotype naming is HLA-A*31:01|HLA-B*51:01|HLA-C*15:02 where P = Present and A = Absent. OR = Odd Ratio PV = logistic p-value; MAF_A=Minor Allele Frequency in cases; MAF_UN=Minor Allele Frequency in controls;

Table S4: Effect size of the association of the top associated Amino acid substitutions in the original SCAR meta-analysis and after conditioning for the top results.

		original				cond 73I				cond A*33:01				cond 73I 80I			
**Marker**	**Allele**	**OR**	**UCI**	**LCI**	**PV**	**OR**	**UCI**	**LCI**	**PV**	**OR**	**UCI**	**LCI**	**PV**	**OR**	**UCI**	**LCI**	**PV**
**AA_A_73_30018729_I**	Isoluecine	5.51	10.37	2.93	1.198E-07	-	-	-	-	-	-	-	-	-	-	-	-
**AA_A_114_30019093_Q**	Glutamine	3.09	5.04	1.89	0.000006	2.00	3.79	1.05	0.03	2.06	3.77	1.13	0.02	1.88	3.58	0.99	0.06
**AA_B_80_31432476_I**	Isoleucine	2.81	4.60	1.71	0.00004	2.71	4.45	1.65	0.00008737	2.62	4.33	1.59	0.0002	-	-	-	-
**AA_A_114_30019093_RH**	Arginine/Histidine	2.53	4.10	1.57	0.0001	1.60	2.96	0.86	0.1	1.67	3.01	0.93	0.09	1.56	2.90	0.84	0.2
**AA_A_56_30018678_R**	Arginine	3.33	6.32	1.76	0.0002	-	-	-	-	0.57	4.24	0.08	0.58	1.13	2.98	0.43	0.80
**AA_A_56_30018678_G**	Glicine	3.31	6.29	1.75	0.0002	-	-	-	-	0.59	3.98	0.09	0.59	1.14	3.01	0.43	0.79
**AA_A_156_30019219_RW**	Arginine/Tryptophan	1.97	3.20	1.21	0.0061	2.60	4.35	1.55	0.0003	2.48	4.14	1.49	0.0005	2.22	3.75	1.32	0.003
**rs116071718**	-	3.32	1.92	5.74	0.00002	3.76	2.14	6.61	0.000004	3.45	1.97	6.01	######	3.52	2.01	6.16	1.09E-05

OR = Odd Ratio; LCI= 95% lower Confidence interval of the Odd Ratio; UCI= 95% upper Confidence Interval of the

Table S5: Effect size of the association of the top associated variants within clinical phenotypes

rs187926838

Clinical Subtypes	OR(LCI-UCI)	PV	MAF Cases
**SJS/TEN**	9.5(2.11-42.98)	0.003	0.06
**DRESS**	18.2(5.20-63.72)	0.000001	0.08
**DILI**			0

HLA-B*51:01

Clinical Subtypes	OR(LCI-UCI)	PV	MAF Cases
**SJS/TEN**	1.48(0.44-4.94)	0.5	0.09
**DRESS**	5.72(2.69-12.16)	$5.7*10^{-6}$	0.14
**DILI**	-	-	0

HLA-B*57:01

Clinical Subtypes	OR(LCI-UCI)	PV	MAF Cases
**SJS/TEN**	6.16 (2.47-15.37)	$9.8*10^{-5}$	0.2
**DRESS phase1***	-	-	0
**DRESS phase2***	1.12 (0.16-8.03)	0.9	0.04
**DILI**	2.4 (0.58-10.27)	0.2	0.08

OR (LCI-UCI) = Odd Ratio (95% Confidence interval of the Odd Ratio); PV = logistic p-value; MAF Cases =Minor Allele Frequency in cases; *We reported both the DRESS phase 1 and DRESS phase 2 associations since they were not concordant.

Table S6: Genotyping details of the control cohorts

COHORT	BATCH	#SAMPLEs	Genotyping CHIP
Welcome Trust Case Control Consor	A	4792	Illumina 1M beadchip
phs000346.v1 (dbGAP)	B	2079	Illumina 1M beadchip
Hypergenes	B	901	Illumina 1M beadchip
LAM30004 collection controls	B	60	Illumina 1M beadchip
Swedish Twin Registry	C	1499	Illumina 1M Duo beadchip
National Spanish DNA Bank	E	173	Illumina 1M Duo/Illumina Infinium HumanCoreExome beadchip
	E	204	Illumina Infinium HumanCoreExome beadchip
iSAEC Italian Penicillin Tolerant Co	D	147	Illumina HumanOmniExpress BeadChip
PGX40001 collection controls	B	103	Illumina 1M Duo beadchip
TSI	B	88	Illumina 1M Duo beadchip
POPulation REference Sample (POP	A	655	Illumina 1M Duo beadchip

References

(1) Price, A.L., Patterson, N.J., Plenge, R.M., Weinblatt, M.E., Shadick, N.A. & Reich, D. Principal components analysis corrects for stratification in genome-wide association studies. . *Nature Genetics* **38**, 904-9 (2006).

(2) Delaneau, O., Zagury, J.F. & Marchini, J. Improved whole-chromosome phasing for disease and population genetic studies. *Nature Methods* **10**, 5-6 (2013).

(3) Marchini, J. & Howie, B. Genotype imputation for genome-wide association studies. *Nature Reviews Genetics* **11**, 499-511 (2010).

(4) Pruim, R.J. *et al.* LocusZoom: regional visualization of genome-wide association scan results. *Bioinformatics* **26**, 2336-7 (2010).
